# MiRNA203 suppresses the expression of protumorigenic STAT1 in glioblastoma to inhibit tumorigenesis

**DOI:** 10.18632/oncotarget.12401

**Published:** 2016-10-02

**Authors:** Chuan He Yang, Yinan Wang, Michelle Sims, Chun Cai, Ping He, Junming Yue, Jinjun Cheng, Frederick A. Boop, Susan R. Pfeffer, Lawrence M. Pfeffer

**Affiliations:** ^1^ Department of Pathology and Laboratory Medicine, University of Tennessee Health Science Center, Memphis, Tennessee; ^2^ Center for Cancer Research, University of Tennessee Health Science Center, Memphis, Tennessee, USA; ^3^ Department of Neurosurgery, University of Tennessee Health Science Center, Memphis, Tennessee, USA; ^4^ Department of Nephrology, Shengjing Hospital of China Medical University, Shenyang, China

**Keywords:** miR203, glioblastoma, tumorigenesis, apoptosis, STAT1

## Abstract

MicroRNAs (miRNAs) play critical roles in regulating cancer cell proliferation, migration, survival and sensitivity to chemotherapy. The potential application of using miRNAs for cancer prognosis holds great promise but miRNAs with predictive value remain to be identified and underlying mechanisms of how they promote or suppress tumorigenesis are not completely understood. Here, we show a strong correlation between miR203 expression and brain cancer patient survival. Low miR203 expression is found in subsets of brain cancer patients, especially glioblastoma. Ectopic miR203 expression in glioblastoma cell lines inhibited cell proliferation and migration, increased sensitivity to apoptosis induced by interferon or temozolomide *in vitro*, and inhibited tumorigenesis *in vivo*. We further show that STAT1 is a direct functional target of miR203, and miR203 level is negatively correlated with STAT1 expression in brain cancer patients. Knockdown of STAT1 expression mimicked the effect of overexpression of miR203 in glioblastoma cell lines, and inhibited cell proliferation and migration, increased sensitivity to apoptosis induced by IFN or temozolomide *in vitro*, and inhibited glioblastoma tumorigenesis *in vivo*. High STAT1 expression significantly correlated with poor survival in brain cancer patients. Mechanistically, we found that enforced miR203 expression in glioblastoma suppressed STAT1 expression directly, as well as that of a number of STAT1 regulated genes. Taken together, our data suggest that miR203 acts as a tumor suppressor in glioblastoma by suppressing the pro-tumorigenic action of STAT1. MiR203 may serve as a predictive biomarker and potential therapeutic target in subsets of cancer patients with low miR203 expression.

## INTRODUCTION

Brain tumors represent an important cause of cancer-related morbidity and mortality in the United States, with malignant gliomas being among the most aggressive and difficult to treat [1]. Although they rarely metastasize, malignant gliomas are locally invasive tumors. The median survival for patients with glioblastoma (GBM), the most common histological subtype of glioma in adults, is ~15 months and has remained this dismal for decades [1]. Adjuvants to surgical resection, including chemotherapy and radiation therapy, currently provide little improvement in the disease course and outcome for GBM patients, and few patients are ever cured [2]. Patients with recurrent GBM have an even bleaker prognosis [3]. Thus, treatment of GBM patients is a significant clinical problem requiring molecular insights into tumor progression and novel therapeutic approaches.

STAT proteins are the downstream effectors of the interferon (IFN) family of cytokines that recognize promoter elements in IFN-stimulated genes (ISGs) to directly activate their transcription [4-6]. IFNs are endogenous antiviral proteins, which also have anticancer activity *in vitro* and have clinical efficacy in the treatment of human cancer, which involves the inhibition of cell proliferation [7] and regulation of cellular responses to inducers of apoptosis [8]. The IFN/STAT1-regulated gene signature predicts poor survival outcomes in certain molecular subtypes of glioblastoma patients [9]. Defects in the IFN system can lead to increased susceptibility to cancer through mechanisms that are incompletely understood [10]. ISGs are inducible by radiation and chemotherapy and regulate therapeutic resistance in preclinical cancer models [11-14]. Interestingly, STAT1 has been reported to have both tumor suppressive [15, 16] and protumorigenic activity [17, 18].

MicroRNAs (miRNAs) are abundant, endogenous, small (20 to 24 nucleotide) single-stranded RNAs that suppress the expression of genes implicated in such fundamental biological processes as differentiation, proliferation and apoptosis [19, 20]. Although individual miRNAs do not encode protein, they control cellular protein expression by the perfect or imperfect binding of their seed sequences to the 3’ untranslated region (UTR) of target mRNAs, promoting their cleavage or inhibiting mRNA translation, respectively [19, 21]. Through loss and gain of function studies, miRNAs have been shown to play key roles in cancer initiation, progression and metastasis [20, 22]. Patterns of miRNA expression are often predictive of tumor classification and prognosis, and miRNAs apparently are involved in regulating the sensitivity of tumor cells to radiation and chemotherapeutic drugs [23].

In general, miRNAs that promote tumorigenesis are overexpressed in cancer, while miRNAs that inhibit the tumorigenic process are underexpressed. For example, miR21 is frequently overexpressed in various human tumors and silences the expression of tumor suppressors; miR21 plays an important role in the oncogenic process and is associated with high proliferation, low apoptosis, high invasion and metastatic potential [24-33]. While oncogenic miRNAs have been studied in depth, tumor suppressor miRNAs which tend to be underexpressed in cancer have been relatively poorly characterized. We recently identified miR203 to have tumor suppressive activity in ovarian cancer by targeting Slug/snail2 and inhibiting epithelial to mesenchymal transition [34]. In the present study we found that miR203 was expressed at especially low levels in GBM. Gene profiling of GBM cell lines with enforced miR203 expression identified STAT1 as a potential miR203 target gene, which was validated by reporter constructs driven by the miR203 binding sequence in 3’UTR of STAT1 mRNA. In addition, enforced miR203 expression in glioma cells silenced STAT1 expression, and decreased the expression of STAT1 regulated genes. Overexpression of miR203 or STAT1 knockdown inhibited the growth of GBM tumors in immunocompromised mice. Furthermore, miR203 expression is inversely related to STAT1 levels in patient samples from glioma, and high STAT1 expression closely correlates with poor patient survival and prognosis. Our findings demonstrate that miR203 has tumor suppressive activity by silencing pro-tumorigenic STAT1. MiR203 is underexpressed, while STAT1 is overexpressed in glioblastoma patients, thereby promoting tumorigenesis.

## RESULTS

### MiR203 expression in GBM patient samples

**Figure 1:**
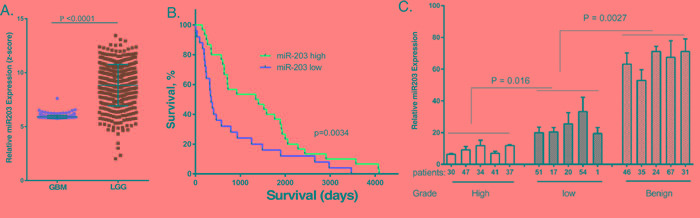
MiR203 expression in cancer patient samples, and the relationship to patient survival **A.** miR203 expression in the TCGA database for glioblastoma (GBM) and low grade glioma (LGG). **B.** miR203 expression in the TCGA database of 60 LGG patient samples with accompanying patient survival information was related to patient survival. **C.** RNA was extracted from five individual de-identified formalin fixed paraffin embedded patient biopsies identified as GBM, LGG or normal brain tissue, and miR203 expression was determined by qPCR (*n* = 3), and normalized to U6A expression [36].

To examine the potential role of miR203 in brain cancer, we examined miR203 expression in patient specimens from low grade glioma (LGG) and the most malignant form of glioma, GBM, in The Human Cancer Genome (TCGA) database. As shown in Figure 1A, miR203 expression was present at consistently low levels in GBM when compared to its expression in LGG. However, it is important to note that in LGG, a subset of patients with relatively low miR203 expression could be discerned. When the LGG patient samples with accompanying survival data in the TCGA database were grouped according to miR203 expression (Figure 1B), high miR203 LGG patients had significantly longer survival (~4-fold) as compared to patients with low miR203 expression (mean of 1370 and 349 days, respectively). To further characterize miR203 expression in brain cancer RNA was isolated from FFPE specimens from five LGG, GBM and histologically normal brain samples and miR203 expression was determined by qPCR. As shown in Figure 1C, there was a statistically significant differences in miR203 expression, with lowest expression in GBM and highest expression in normal brain tissue.

### The effects of enforced miR203 expression on glioma cell proliferation, migration and sensitivity to induction of apoptosis

**Figure 2:**
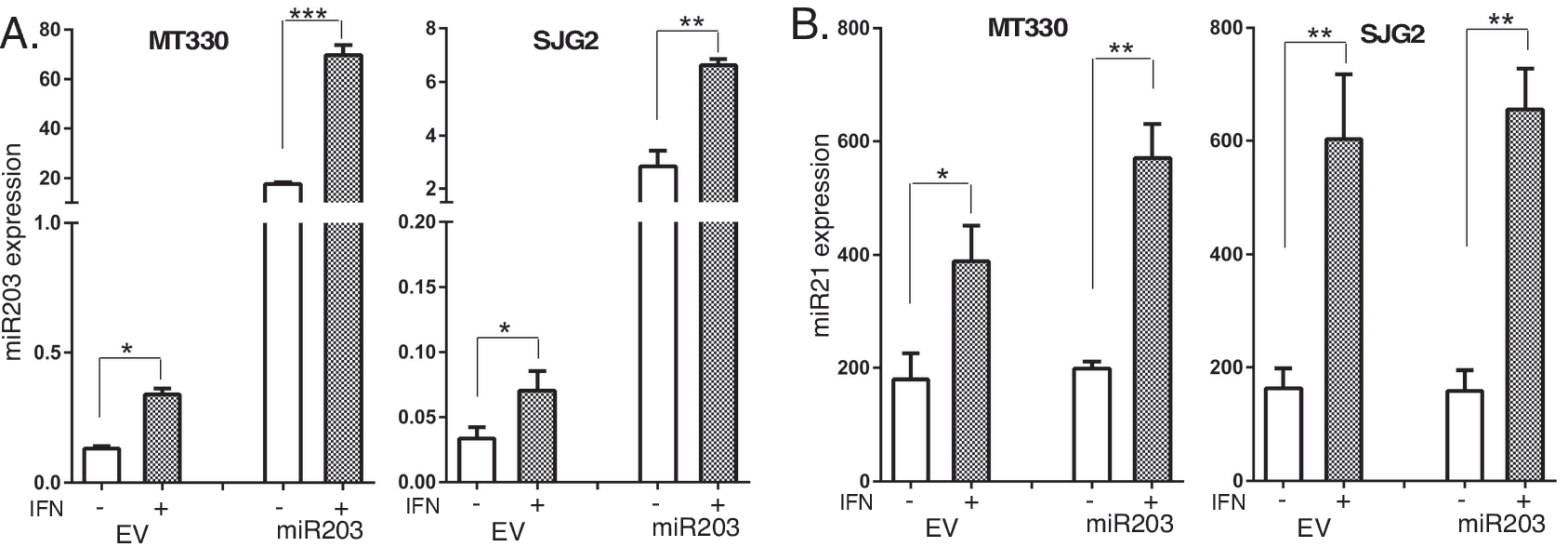
IFN induces miR203 expression **A.**, **B.** Total RNA was prepared from empty vector (EV) and miR203 enforced GBM cell lines, which were not treated (con) or treated with IFN (1,000 IU/ml for 6 hr), and the expression of miR203 (A) and miR21(B) determined by qPCR.

**Figure 3:**
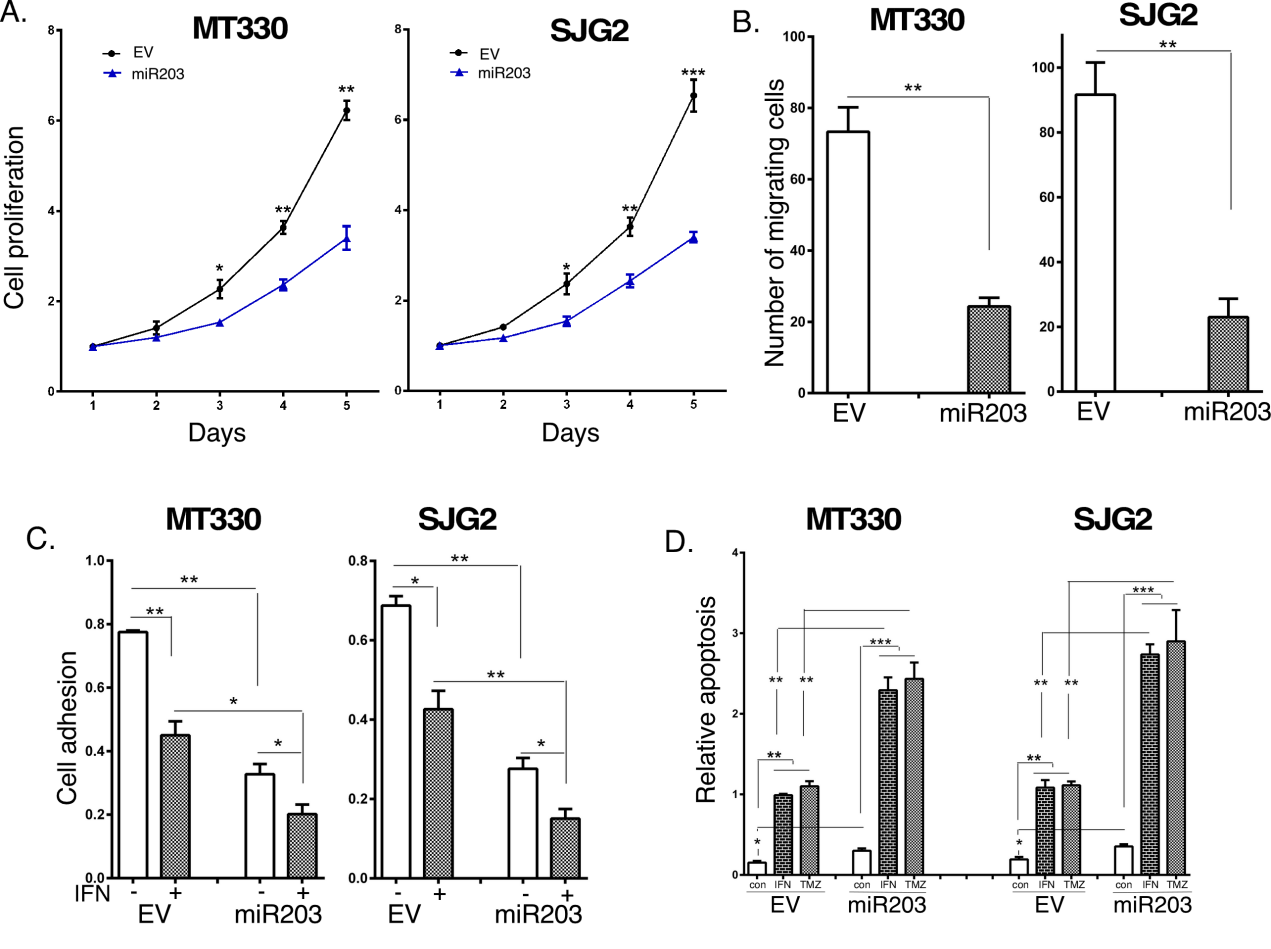
The effects of Enforced miR203 expression on glioma cell proliferation, migration and sensitivity to induction of apoptosis Cell proliferation **A.** migration **B.** and fibronectin cell adhesion **C.** of EV and miR203-enforced GBM cells. The effect on cell adhesion was also measured in the presence or absence of IFN treatment (1,000 IU/ml for 24 hr). **D.** EV and miR203-enforced GBM cells were treated for 24 hr with IFN (1,000 IU/ml) or TMZ (100 μM) and analyzed for apoptosis by cell death detection ELISA assays.

Since miR203 was underexpressed in GBM, we examined the biological consequences of enforced miR203 expression in MT330 and SJG2 human GBM cell lines with low basal miR203 expression. Cells were transduced with miR203-encoding lentivirus, and stable pools were isolated after puromycin selection. Since miR203 was reported to be IFN-inducible [35], we also treated GBM cells with IFN, isolated RNA and determined miR203 expression by quantitative real-time PCR (qPCR). Both MT330 and SJG2 transduced GBM cells have significantly higher miR203 expression than empty vector (EV)-transduced cells, and IFN induced an increase in miR203 expression in both EV and miR203 expressing cells (Figure 2A). Moreover, while IFN-induced miR21 expression in both GBM cell lines (Figure 2B) as we have previously shown [36-40], enforced miR203 expression had no effect on miR21 expression (Figure 2C). The effect of enforced miR203 expression on the proliferation of EV and miR203-transduced GBM cells was monitored daily by cell counting, and found to inhibit cell proliferation (Figure 3A). By cell migration assays, we found that enforced miR203 expression also inhibited cell migration (Figure 3B). Since cell adhesion is a complex process involved in cell migration/invasion, we also performed fibronectin adhesion assays and found that enforced miR203 expression as well as IFN treatment markedly reduced cell adhesion, and together they had an even greater effect on cell adhesion (Figure 3C). Since we previously found that apoptosis in cancer cell lines is counterbalanced by a miRNA-regulated cell survival pathway [36], apoptosis in GBM cells with enforced miR203 expression was determined by a cell-death ELISA. Cells were treated with IFN or temozolomide (TMZ), the frontline chemotherapy for GBM that induces apoptosis through DNA strand breaks [41]. While basal apoptosis was slightly increased by enforced miR203 expression, apoptosis induced by IFN and TMZ was markedly increased in both MT330 and SJG2 cells with enforced miR203 expression (Figure 3D). These results taken together are consistent with the hypothesis that miR203 inhibits GBM cell proliferation and cell migration and adhesion, and enhances sensitivity of GBM cells to inducers of apoptosis.

### Identification of STAT1 as a miR203 target gene

**Figure 4:**
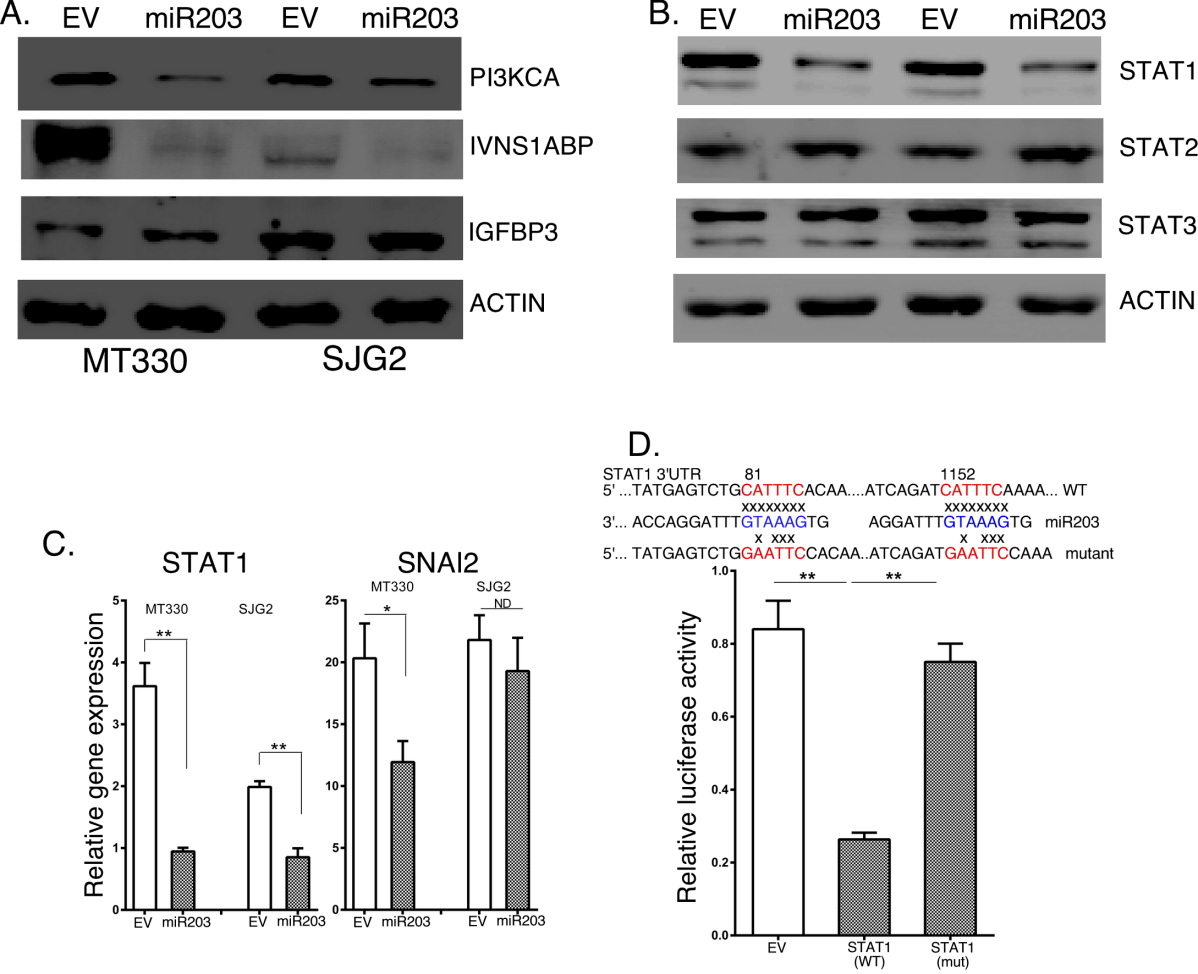
The effects of enforced miR203 expression on target genes **A.** Lysates were prepared from EV and miR203 enforced GBM cells and immunoblotted as indicated. **B.** RNA was extracted from EV and miR203 enforced GBM cells, and gene expression determined by qPCR, and normalized to actin expression (*n* = 3). **C.** Upper Panel: Sequence alignment of the miR203 binding sequence with the 3’ UTR of STAT1 gene (wild-type) and a mutant STAT1 construct used for experiments. Lower Panel: 293T cells were transiently cotransfected with empty-vector (pcDNA3.1-Luc), or wild-type (pcDNA3.1-Luc-wtUTR) or mutant (pcDNA3.1-Luc-muUTR) STAT1 reporter plasmids, and with miR203 plasmid. pSV40-Renilla plasmid was cotransfected as an internal control. The ratio of luciferase and Renilla activities was determined at 24 h post-transfection using the dual luciferase reporter gene kit (Promega).

MiRNAs control cellular protein expression by binding to target mRNAs and silencing their expression. To identify potential miR203 targets, we isolated RNA from MT330 and SJG2 cells with enforced miR203 expression, performed microarray analysis, and found that, although the expression of several hundred genes were down-regulated by enforced miR203 expression, the expression of STAT1, IVNS1ABP and PI3KCA were consistently downregulated in both GBM cell lines. Most interesting is that, while IVNS1ABP and PI3KCA were identified by bioinformatics algorithms such as TargetScan as having miR203 binding sites in their 3’UTR, STAT1 was not identified by this analysis. To determine whether these genes were silenced by enforced miR203 expression, whole cell lysates were prepared from EV and miR203 transduced GBM cells, and protein expression determined by immunoblotting. As shown in Figure 4A, while the levels of STAT1, PI3KCA and IVNS1ABP were reduced in GBM cells with enforced miR203 expression, expression of the miR21 target gene IGFBP3 was unaffected by miR203 expression. We focused our analysis on STAT1 as a miR203 target gene because the role of STAT1 in IFN signaling, and STAT1 has been reported to have both tumor suppressive [15, 16], and protumorigenic activity [17, 18]. To determine whether expression of STAT proteins was globally suppressed by miR203 expression, whole cell lysates were also immunoblotted for different STATs. While STAT1 expression is markedly lower in cells with enforced miR203 expression, both STAT2 and STAT3 are unaffected by miR203 expression (Figure 4A). Moreover, while STAT1 was a miR203 target gene in GBM cells, as shown in Figure 4B Snai2/Slug was not consistently silenced by miR203 expression in GBM cells, although it was targeted by miR203 in ovarian cancer cells [34]. This finding is also consistent with our previous findings that miRNAs silence gene expression in a cell-type specific manner.

Using the miR203 core seed sequence (GUAAAGU) we identified two potential miR203 binding sites of CAUUUCA in the 3’ UTR of STAT1 (Figure 4C). To determine whether STAT1 was a direct miR203 target, the 3’UTR of STAT1 mRNA containing the two predicted miR203 target sequences as well as a corresponding mutated sequence were linked to luciferase, and a dual-luciferase (pcDNA3.1-Luc) reporter system was employed to evaluate miRNA:mRNA interactions. Overexpression of miR203 in HEK293T cells downregulated luciferase activity of the STAT1-driven wild-type reporter constructs, while luciferase constructs with two mutated miR203 binding sequences in STAT1 were unaffected by miR203 overexpression (Figure 4C). These results show that STAT1 is a bona fide miR203 target gene.

### The effects of STAT1 knockdown on glioma cell proliferation, migration and sensitivity to induction of apoptosis

**Figure 5:**
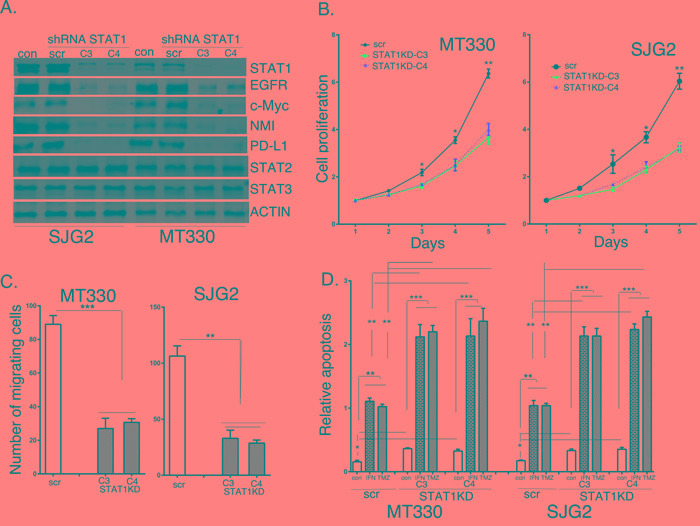
The effects of STAT1KD on STAT1 target gene expression and glioma cell proliferation, migration and sensitivity to induction of apoptosis **A.** Lysates were prepared from GBM cells transduced with scrambled or shRNAs directed against two different regions of STAT1, and immunoblotted as indicated. Cell proliferation **B.**, migration **C.**, and apoptosis **D.** of scrambled shRNA transduced (scr) and STAT1KD GBM cells. In apoptosis experiments GBM cells were treated for 24 hr with IFN (1,000 IU/ml), and analyzed for apoptosis by cell death detection ELISA assays.

To characterize the functional role of STAT1 we knocked down (KD) STAT1 expression in GBM cell lines. In brief, MT330 and SJG2 cells were transduced with several STAT1 short hairpin RNA (shRNA)-miR lentiviral vector pGIPZ, which contain miR30 hairpin-based sh-miR structure against different regions of STAT1 (C3 and C4). Transduced cells were selected with puromycin, and after selection maintained without puromycin. Cell lysates were prepared, and immunoblotted with Abs against STATs and various STAT1-regulated proteins. As shown in Figure 5A, while the scrambled shRNA control had no effect on the levels of any of the proteins examined, both C3 and C4 STAT1-shRNAs inhibited the expression of STAT1 and known STAT1 regulated genes, including Epidermal Growth Factor Receptor (EGFR), c-Myc, N-Myc inhibitor (NMI), and programmed death ligand 1 (PD-L1). The selectivity of the STAT1KD was further supported by the findings that STAT2 and STAT3 expression was unaffected by STAT1KD. Similar results on gene expression were obtained by qPCR on RNA extracts of these cells. We then examined the effect of STAT1KD on the proliferation of MT330 and SJG2 glioma cells, and found that similar to the results with enforced miR203 expression STAT1KD resulted in a decrease in cell proliferation (Figure 5B) and cell migration (Figure 5C). We next compared basal apoptosis and apoptosis induced by IFN and TMZ in STAT1KD GBM cells. As shown in Figure 5D, while basal apoptosis was only slightly affected by STAT1KD, apoptosis induced by IFN and TMZ was markedly increased in both MT330 and SJG2 cells with STAT1KD. Taken together these results are consistent with the hypothesis that STAT1KD inhibits GBM cell proliferation, inhibits GBM cell migration and adhesion, and enhances sensitivity of GBM cells to inducers of apoptosis, and thus mirrors the effect of miR203 expression consistent with STAT1 being a miR203 target gene.

### Enforced miR203 expression and STAT1 knockdown inhibits glioma tumor growth *in vivo*

**Figure 6:**
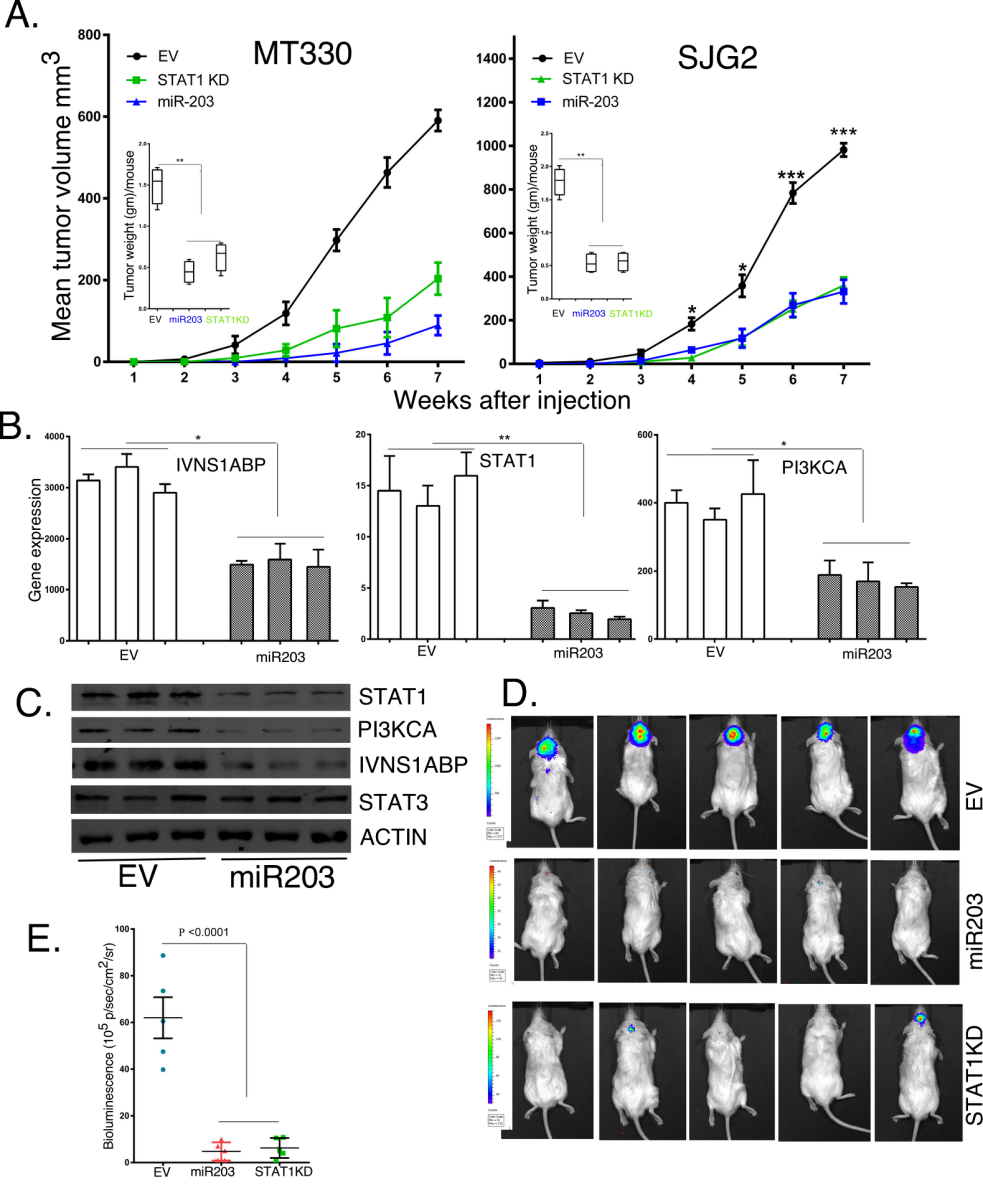
The effects of enforced miR203 expression and STAT1KD on tumor formation by GBM cell lines **A.** NSG mice were injected subcutaneously with 10^6^ EV, miR203 enforced or STAT1KD-C4 GBM cells, and tumor growth was determined by caliper measurements. Inset: weight of tumors at necropsy. Tumors were extracted from mice injected with EV or miR203 enforced SJG2 cells and assessed for RNA **B.** and protein **C.** levels of IVNS1ABP, STAT1 and PI3KA by qPCR and immunoblotting, respectively. **D.** Five NSG mice were injected intracranially with 10^6^ EV, miR203 enforced or STAT1KD-C4 GBM cells, and tumor growth was determined by live animal imaging at 6 weeks. **E.** Quantification of the bioluminescent signal of injected mice.

We then sought to determine the roles of miR203 and STAT1 in the tumorigenicity of GBM cells *in vivo*. NSG mice were injected subcutaneously with MT330 and SJG2 GBM cells with enforced miR203 expression or STAT1KD, and tumor volume was determined by caliper measurement. Enforced miR203 expression or STAT1KD markedly suppressed tumor formation by both GBM cell lines (Figure 6A). Moreover, when tumors were weighed at six to seven weeks after injection, there was marked reduction in tumor weight in mice injected with GBM cells with miR203 enforced expression or STAT1KD. To validate that the inhibition of tumor formation reflected enforced miR203 expression, RNA and protein was extracted from three individual tumors of mice injected with EV and miR203 expressing SJG2 cells. Expression of the miR203 target genes, STAT1, PI3KCA and IVNS1ABP, was markedly inhibited in tumor extracts from mice injected with enforced miR203 expressing cells as determined by qPCR (Figure 6B), and immunoblotting (Figure 6C). Tumor tissue from mice injected with GBM cells with enforced miR203 expression had lower protein levels of miR203 targets (STAT1, PI3KCA and IVNS1ABP), but the levels of STAT3 and actin were unaffected. Furthermore, the effect of enforced miR203 expression or STAT1KD was also determined in the orthotopic (brain) microenvironment for GBM by intracranial injections of luciferase-expressing cells, and tumorigenesis followed by live animal imaging after D-luciferin injection. Although EV transduced MT330 exhibited significant bioluminescent signal throughout the brain of injected mice demonstrating tumor invasion, enforced miR203 expression or STAT1KD resulted in a marked reduction in bioluminescent signal which is localized around the injection site after intracranial injection of luciferase labeled cells (Figure 6D).

### STAT1 expression correlates with poor overall patient survival in GBM

**Figure 7:**
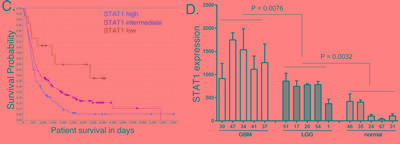
STAT1 expression in cancer patient samples, and the relationship to patient survival **A.** STAT1 expression in the TCGA database for GBM patients was plotted against patient survival. **B.** GBM patients in the TCGA database with the lowest and highest survival were compared for STAT1 expression. **C.** Kaplan-Meier analysis of the survival of glioma patients in the REMBRANT database grouped according to relative STAT1 expression. **D.** RNA was extracted from five de-identified FFPE patient biopsies identified as GBM, LGG or normal brain tissue, and STAT1 expression was determined by qPCR (*n* = 3), and normalized to actin expression [36].

We next examined the relationship between STAT1 expression to patient survival. In GBM samples in the TCGA database we found a statistically significant inverse relationship between STAT1 expression and patient survival using the Pearson correlation (Figure 7A), showing that high STAT1 expression is associated with poor patient survival (*p* < 0.001). Furthermore, if GBM patients are grouped into the top 10% and lowest 10% survival, the patients with the lowest overall survival have significantly higher STAT1 expression (*p* < 0.001) (Figure 7B). We next examined individual patient survival in the Repository of Molecular Brain Neoplasia Data (REMBRANT) for glioma patients. As shown in Figure 7C, patients bearing STAT1-high glioma had significantly shorter survival than those bearing STAT1-low glioma (*p* = 0.0009). Moreover, patients bearing STAT1-intermediate glioma have longer disease-specific survival when compared with STAT1-high glioma (*p* = 0.0023), and shorter disease-specific survival when compared with STAT-low glioma (*p* = 0.0279) (Figure 7C). To further characterize STAT1 expression in brain cancer, RNA was isolated from FFPE biopsy specimens from five LGG, GBM and histologically benign brain samples and STAT1 expression was determined by qPCR. As shown in Figure 7D, there were statistically significant differences in STAT1 expression with highest expression in GBM and lowest expression in benign brain tissue. Moreover, in the same patient specimens the expression of STAT1 (Figure 7D) and miR203 (Figure 1C) are inversely related.

## DISCUSSION

GBM is the most common primary brain malignancy and carries the worst prognosis. Despite improved molecular characterization of the disease, aggressive surgery, radiation, and chemotherapy are standard of care in GBM, and the median survival of GBM patients still remains only 12- to 15-months. Thus, identification of new molecular targets in GBM may lead to improved therapeutic approaches. In the present study, we found that miR203 was nearly universally expressed at extremely low levels in GBM patient samples in the TCGA database However, in the more benign form of brain cancer LGG, which overall has a better patient prognosis, there is a significant variation in miR203 expression, and we found that high miR203-expressing patients had significantly 4-fold longer survival than low miR203-expressing patients. These results suggest that miR203 may have value as a prognostic biomarker in brain cancer.

Moreover, we found that *in vitro* enforced miR203 expression inhibited GBM cell proliferation and migration, and increased sensitivity to apoptosis induced by the cytokine IFN and the chemotherapeutic agent TMZ, both of which have been used to treat GBM in the clinic. In addition, the tumorigenicity *in vivo* of GBM cells with enforced miR203 expression was markedly impaired when injected subcutaneously or intracranially into immunocompromised mice. Taken together these results indicate that miR203 may have tumor suppressive function in GBM. These findings are consistent with our previous study in ovarian cell lines, which show that miR203 has tumor suppressive function [34]. However, although Snai2/Slug is an important target of miR203 in ovarian cancer, we found that Snai2/Slug expression was not consistently suppressed by enforced miR203 expression in GBM cell lines. To identify new miR203 target genes we performed gene expression analysis on RNA samples of control and miR203-enforced GBM cell lines, and identified that STAT1 as a potential miR203 target gene. By luciferase reporter assays driven by wild-type and mutant 3’UTR of STAT1, we showed that STAT1 expression was directly regulated by miR203 expression. Our finding that STAT1 is a miR203 target gene is particularly interesting because STAT1 has been reported to have both tumor suppressive [15, 16], and protumorigenic activity [17, 18]. We hypothesized that STAT1 would be protumorigenic in glioma, which was consistent with our finding that STAT1 was expressed at high levels in GBM cells lines. Therefore, we knocked down STAT1 in GBM cell lines and found that this mimicked the effects of enforced miR203 expression. STAT1KD GBM cell lines showed inhibited proliferation and migration when compared to cells transduced with empty vector, as well as increased sensitivity to apoptosis induced by IFN and TMZ. Moreover, STAT1KD GBM cell lines showed diminished tumorigenicity when injected into immunocompromised mice. Taken together these results suggest that STAT1 promotes tumorigenicity in GBM. Interestingly, STAT1KD inhibited the expression of several known STAT1 regulated genes, including EGFR, c-Myc, NMI, and PD-L1. All of these genes are known to play important roles in the tumorigenicity of GBM. For example, high NMI expression has been found to predict poor prognosis and promotes tumor growth in human GBM [42]. High PD-L1 expression is correlated with poor outcome in GBM [43]. Dysregulation of the epidermal growth factor receptor (EFGR) is found in ~50% of GBM patient samples analyzed [44, 45]. In gliomas, c-Myc expression has been found to correlate with the grade of malignancy in brain cancer, with low expression in LGG and high expression in GBM [46].

We also examined the relationship between STAT1 expression in publically available databases on patient survival in glioma. We found that low STAT1-expressing glioma patients had the best overall survival when compared to high STAT1-expressing patients, with intermediate patients falling somewhere in between in overall survival. Moreover, when we examined GBM patients specifically, those with the worst overall survival had significantly higher STAT1 levels when compared to GBM patients with lowest STAT1 expression. These results suggest that high STAT1 expression predicts poor prognosis in GBM. Consistent with our findings an expression signature of IFN/STAT1 signaling genes was found to predict poor survival outcome in GBM patients [9].

In addition, there are clearly other forms of cancer besides glioma where the miR203/STAT1 pathway may play an important role. MiR203 expression in TCGA database was present at consistently low levels in GBM when compared to its expression in cancers of the ovary (OV), skin (SK), liver (HC), prostate (PR), cervix (CE) and breast (BR), as well as LGG (Figure S1). However, in nearly all the cancers examined, except prostate, breast and cervical cancer, a subset of patients with relatively low miR203 expression could be discerned. Moreover, in liver cancer both high miR203-expressing and low STAT1-expressing patients had a significantly longer survival than low miR203 expressing patients and high STAT1-expressing patients (Figure S2). Similarly, we previously showed that in ovarian cancer miR203 expression is significantly higher in patients with the best overall survival when compared to patients with the worst overall survival [34]. Thus, there are subsets of cancer patients with low miR203 expression for whom miR203 targeted therapies may have therapeutic benefit. The downregulation of a tumor-suppressive miRNA in cancer such as miR203 may be overcome by introducing synthetic oligonucleotides that are identical to the miRNA, known as miRNA mimics.

In summary, we identified STAT1 as a novel miR203 target gene in GBM, and characterized the role of the opposing actions of miR203 and STAT1 in GBM as well as in biopsy specimens from cancer patients, which suggests that miR203 inhibits GBM tumorigenesis through suppressing the expression of the tumor promoting activity of STAT1.

## MATERIALS AND METHODS

### Biological reagents and cell cultures

The biological activity of recombinant human interferon (IFNcon1, InterMune) was expressed in terms of international reference units/ml using the human NIH reference standard [47]. Antibodies against the following proteins were used: STAT1, STAT2, PTEN, IVNS1ABP, PI3KCA, EGFR, c-Myc, NMI, PD-L1 and actin (Santa Cruz Biotechnology); STAT3 (BD Transduction Laboratories); PDCD4, (Abcam) and FBXO11 (Novus Biologicals). MT330 (UTHSC Department of Neurosurgery), and SJG2 (St. Jude Children's Research Hospital) cell lines were grown in DMEM containing 10% Fetal Bovine serum (Atlanta Biologics) supplemented with penicillin (100 IU/ml) and streptomycin (100 mg/ml) at 37 °C with 5% CO_2_.

### Gene expression analysis

Total RNA was isolated using RNeasy Mini kit (Qiagen) from empty vector and miR203 expressing MT330 and SJG2 cells, and submitted to the UTHSC Center of Genomics and Bioinformatics (Memphis, TN) for labeling and hybridization to Human-HT12 BeadChips (Illumina). Microarray data analysis was then carried out using GenomeStudio 3.4.0 and GeneSpring software 7.0 (Silicon Genetics), and expression values for each gene were normalized as described previously [48]. The average fold-change in gene expression from three independent sets of GeneChip data was subjected to non-parametric *t* testing. For quantitative real time PCR (qPCR) analysis, RNA was isolated using the RNeasy Mini kit (Qiagen), and gene expression was determined as previously described [36], using the following gene-specific primers: Snai2, 5’- CGAACTGGACACACATACAGTG-3’ (forward) and 5’- CTGAGGATCTCTGGTTGTGGT-3’ (reverse); IVNS1ABP, 5’- GATGTTCGACTTCAGGTCTGTG-3’ (forward) and 5’- CGTGAGAAATTCCATGAGGATCA-3’ (reverse); STAT1, 5’- CAGCTTGACTCAAAATTCCTGGA-3’ (forward) and 5’- TGAAGATTACGCTTGCTTTTCCT-3’ (reverse); PIK3ca, 5’- CCACGACCATCATCAGGTGAA-3’ (forward) and 5’- CCTCACGGAGGCATTCTAAAGT-3’ (reverse); ACTB 5’- GGACTTCGAGCAAGAGATGG-3’ (forward) and 5’- AGCACTGTGTTGGCGTACAG-3’ (reverse). For miRNA expression, total RNA (5 μg) was reverse-transcribed into first-strand cDNA and 30 ng of cDNA was used as a template for the PCR reaction with a forward primer specific to the mature miR203 sequence, and the following mature miR203 sequence (5’- GTGAAATGTTTAGGACCACTAG-3’). SYBR Green-based real-time PCR was performed on a BioRad iCycler and gene expression normalized relative to U6 or β-actin expression for miRNA or mRNA, respectively. In addition, 3-5 (10 micron) curls were cut from de-identified formalin-fixed paraffin-embedded (FFPE) patient biopsy specimens (UTHSC Tissue Services Core), RNA isolated using the RecoverAll^TM^ Total Nucleic Acid Isolation Kit (Ambion), and gene expression determined by qPCR.

### Lentiviral overexpression of miR203 and STAT1 knockdown

Hsa-miR203 lentivector was purchased from ABM, packaged in HEK293FT cells and produced as previously described [36]. To knockdown STAT1 expression, cells were transduced with the STAT1 short hairpin RNA (shRNA)-miR lentiviral vectors pGIPZ, which contains a miR30 hairpin-based shRNA-miR structures against STAT1 (OPEN Biosystems). Transduced cells were selected with puromycin, and after selection stable pools with expression levels knocked down by >75% or overexpressed by >3-fold were maintained in growth medium without puromycin.

### Immunoblot analysis

Total cell lysates (25 μg) were separated by SDS-PAGE, transferred to polyvinylidene difluoride membranes (Millipore) and immunoblotted with the indicated antibodies, followed by IRDye800CW goat anti-mouse IgG or IRDye680 goat anti-rabbit IgG (LI-COR Biosciences). Blots were visualized on an Odyssey Infrared Imaging System (LI-COR Biosciences).

### Construction of luciferase reporter gene plasmids and reporter assays

We have identified two miR203 binding sites present in the 3’UTR of STAT1. The 3’ UTR of STAT1 containing these predicted binding sites was amplified by PCR from genomic DNA of human 293T cells. After digestion with XhoI and BamHI, the PCR product was purified and cloned into pcDNA3.1-luc, resulting in the wild-type FBXO11 reporter plasmid, pcDNA3.1-Luc-wtUTR. The mutant STAT1 reporter plasmid pcDNA3.1-Luc-muUTR was constructed by mutating the two miR203 binding sites in the 3’UTR of STAT1 using PCR based site-directed mutagenesis according to manufacturer's instructions (Stratagene). The primers for amplifying the wild-type 3’-UTR are 5’- GATACTCGAGCATTGCAAGTATCTTCCTAC -3’ and 5’- GCGGATCCTCAGATTGTATGCAGTGCCA -3’, and the primers for mutant construct are 5’- GAATTCGCAAGATCAGTGTAATAAACTTAACCAC -3’ and 5’- CAGACTCATACATTAAACATTGCTTGTCTATTC -3’ (site 81); 5’- AATTCGCAACTCATTTCCTATGTAACTGCATTGA-3’ and 5’- ATCTGATTCTCATATTATCTCTGGTGTATTA-3’ (site 1152). Reporter gene binding assays were performed by co-transfection of 293T cells using wild-type and mutant reporter plasmids pcDNA3.1-Luc-wtUTR and pcDNA3.1-Luc-muUTR with miR203 overexpressing plasmid, respectively. pSV40-Renilla plasmid was co-transfected as an internal control. The ratio of luciferase and Renilla activities was determined at 24h post-transfection using the dual luciferase reporter gene kit (Promega).

### Tumor formation in mice

Animal experiments were performed in accordance with a study protocol approved by the Institutional Animal Care and Use Committee of the University of Tennessee Health Science Center. Xenografts were established in five-week-old male NOD.Cg-*Prkdc^scid^* *Il2rg^tm1Wjl^*/SzJmice (Jackson Laboratory) by injection of MT330 and SJG2 cells (1x10^6^) directly into the flanks [37]. Tumors were measured weekly with a handheld caliper. In addition, luciferase-expressing GBM cells (10^6^) were injected stereotactically into the superficial brain parenchyma of NSG mice through a burr hole in the skull as previously described [49]. NSG mice were injected with D-luciferin and subjected to live animal imaging weekly to quantify bioluminescence [38, 49].

### TCGA data query

To examine the relationship between miR203 and STAT1 expression in human cancer specimens, we queried the TCGA data portal for all samples with Level 3 miRNA or mRNA expression data available, as well as the accompanying clinical data. The data set was filtered for samples having expression data for miR203, STAT1 and clinical data, yielding a final set of 420 GBM, and 438 LGG cancer patient samples. Statistical analyses were performed using Graphpad Prism.

### Cell proliferative assays

For cell proliferation analysis, cultures were plated at 1 x 10^5^ cells in 25-cm^2^ flasks, and at daily intervals cells were harvested by trypsinization and enumerated in a Coulter Counter [50].

### Transwell migration assays

Cell were subjected to transwell migration assays as previously described [36].

### Apoptosis assays

The induction of apoptosis was monitored by DNA fragmentation using the cell death detection ELISA^PLUS^, according to the manufacturer's protocol.

### Statistical analyses

At least two independent experiments were performed in duplicate, and data are presented as means ± sd. ANOVA and post-hoc least significant difference analysis or Student's t-tests were performed. *p* values < 0.05 (*), 0.01 (**) and 0.001 (***) were considered statistically significant.

## SUPPLEMENTARY MATERIALS FIGURES AND TABLES


