# Supplementary material for: MiRNA203 suppresses the expression of protumorigenic STAT1 in glioblastoma to inhibit tumorigenesis
Source: Oncotarget. 2016 Oct 2;7(51):84017–29. doi: 10.18632/oncotarget.12401 (PMC5341291; doi:10.18632/oncotarget.12401)
Supplement: Supplementary file 1 [file oncotarget-07-84017-s001.pdf]

# MiRNA203 suppresses the expression of protumorigenic STAT1 in glioblastoma to inhibit tumorigenesis

**Supplementary Material**

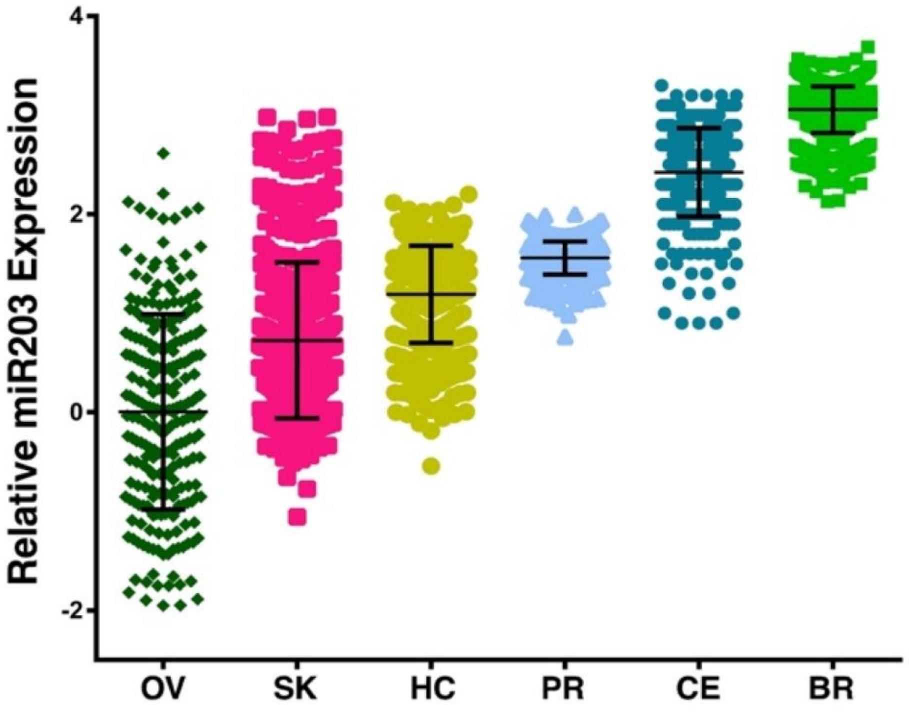


**Figure S1. MiR203 expression in cancer patient samples.** miR203 expression in the TCGA database for cancers of the ovary (OV), skin (SK), liver (HC), prostate (PR), cervix (CE) and breast (BR).

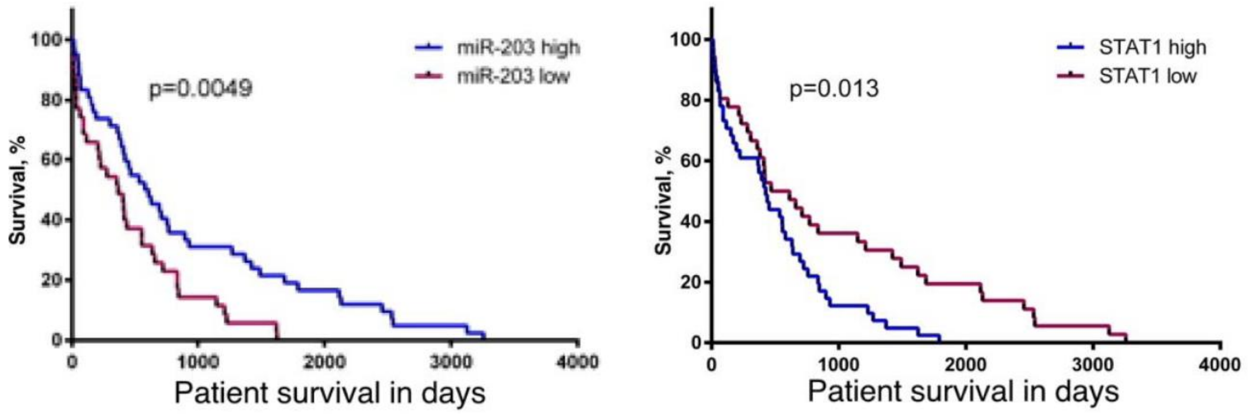


**Figure S2. STAT1 expression in liver cancer patient samples, and the relationship to patient survival.** MiR203 and STAT1 expression in the TCGA database for HC patients was plotted against patient survival.
